# Anæsthesia as Produced by Schleich, of Berlin

**Published:** 1896-04

**Authors:** George Tully Vaughan

**Affiliations:** Philadelphia, Pa. (Passed Assistant Surgeon U.S. Marine Hospital Service.)


ARTICLE VI.

### ANÆSTHESIA AS PRODUCED BY SCHLEICH, OF BERLIN.

BY GEORGE TULLY VAUGHAN, M. D., PHILADELPHIA, PA.
(Passed Assistant Surgeon U. S. Marine Hospital Service.)

Schleich's method of producing local anæsthesia has been somewhat discussed in the medical journals and societies of this country, and with considerable difference of opinion as to its efficiency, mainly, I believe, owing to failure to follow strictly the details as given by the author. In January, 1895, I visited Dr. Schleich in Berlin and saw him operate, using his method of producing local anæsthesia. The solution is:

| | | |
|---|---|---|
| R.—Cocaini muriatis, | - - | 1.00 |
| Morphiæ muriatis, | - | .25 |
| Sodii muriatis, | - - | 2.00 |
| Aquæ Distillat | - - | 1000.00—M. |

The maximum dose, as a rule, is 50 c. c. of this solution, containing .05 grain (¾ grains) of cocaine, which is injected into the tissues to be operated on by means of a hypodermic syringe as follows:

If desired, the slight pain caused by the first insertion of the needle may be prevented by chilling with ether spray. This is not usually necessary. The needle is then inserted *into*—not beneath—the skin and a drop or two of fluid forced out, forming a white wheal in the skin which is instantly deprived of sensation as far as the wheal extends, and a little beyond its borders. The needle is then pushed on from the borders of the first wheal a short distance and a drop or two more injected and so on successively along the line of the intended incision—first in the skin itself, then in the tissues beneath, layer by layer, including periosteum if necessary, until the entire field of cperation is distended with the solution.

Dr. Schleich uses this solution in all minor operations, as opening of abscesses, whitlows, etc.; also in some laparotomies, if there are not too many adhesions, which would requird too large a quantity of the solution for thorough saturation of the tissues. He had used it in cholecystotomy, ventral fixation of the uterus, and in one case of amputation of the forearm. In the last operation there was some pain, but it was slight.

I have used this method in four cases with satisfactory results. In two of them the anæsthesia was not complete; but this, I think, was due to want of sufficient quantity of the solution.

The first case was excision on August 14, 1895, of a fibroma, about the size of a pigeon's egg, from the upper lip of a man. Less than 5 c. c. gave complete anæsthesia.

The second case was circumcision, August 22d, 3 c. c. being used which failed to produce complete insensibility, though the pain was much diminished.

The third case was operated on December 4th, excision of suppurating inguinal glands, and 8 c. c. of the solution were used. The tissues were thickened and inflamed so that the glands lay one to one and a half centimetres from the surface. Incisions with the knife caused no pain, but the tearing of the tissues with the fingers in dissecting out the glands caused a little.

In the fourth the solution was used to prevent pain in aspirating the pericardium; 1.75 c. c. of the solution being injected into the skin and underlying tissues down nearly to the pericardium over the sixth intercostal space. Absolutely no pain was felt on introducing the aspirating needle until the pericardium was punctured.

It is evident that this method of producing local anæsthesia, being so safe and so simple, has a wide field of usefulness, not only in minor, but in certain cases of major operations.

Parke, Davis & Co. now manufacture a tablet, so that this solution can be prepared in a few moments, simply by dissolving a tablet in the proper quantity of water.

Dr. Schleich has also been experimenting with general anæsthetics, especially with three mixtures in different proportions, of benzine, chloroform, and ether, as shown below.

| | | | |
|---|---|---|---|
| R.—Ætheris petroli (benzine), | 5 | 5 | 5 |
| Chloroform, - | 15 | 15 | 30 |
| Ætheris sulphurici, | 60 | 50 | 80 |
| Boil respectively at (centigrade), | 30° | 40° | 42° C. |

The mixture boiling at the lowest temperature (39°) gives the lightest narcosis, and the one boiling at the highest, the deepest.

The advantages claimed are quickness of action sooner recovery from unconsciousness, and that little is left in the system for elimination by lungs and kidneys.

The one patient to whom I saw the lighter mixture given acted as I have often seen those who had taken chloroform. There was no stage of excitement and the patient vomited a little on waking.

Dr. Schleich uses a mixture of marble dust, lysol and wax for cleansing the hands and making them aseptic. Culture medio inoculated with scrapings from beneath the finger nails after cleansing with this mixture, remain sterile. As an ointment and protective to wounds, he uses a powder of oxide of zinc and sterilized blood serum, dried and pulverized, wetting the powder and making a paste before applying. It dries and makes a protective dressing somewhat like collodion—*Virginia Medical Monthly.*

